# Mechanical Clinching and Self-Pierce Riveting of Thin Three Sheets of 5000 Series Aluminium Alloy and 980 MPa Grade Cold Rolled Ultra-High Strength Steel

**DOI:** 10.3390/ma13214741

**Published:** 2020-10-23

**Authors:** Yohei Abe, Takato Maeda, Daiki Yoshioka, Ken-ichiro Mori

**Affiliations:** Faculty of Engineering, Toyohashi University of Technology, 1-1 Tempaku-cho, Toyohashi, Aichi 441-8580, Japan; maeda@plast.me.tut.ac.jp (T.M.); yoshioka@plast.me.tut.ac.jp (D.Y.); mori@plast.me.tut.ac.jp (K.-i.M.)

**Keywords:** joining, mechanical clinching, self-pierce riveting, ultra-high strength steel sheets, aluminium alloy sheet, three sheet, plastic deformation, joint strength

## Abstract

One thin 5000 series aluminium alloy sheet and two thin 980 MPa grade cold rolled ultra-high strength steel sheets were joined by self-pierce riveting and mechanical clinching processes. The joinabilities for a combination of the aluminium and steel sheets in both processes were investigated for different die shapes in the experiment and finite element simulation. In self-pierce riveting, the three sheets were successfully joined for both combinations of the upper and lower aluminium alloy sheets by optimizing the shapes of a die and rivet. In mechanical clinching, the three sheets were successfully joined by an optimum die for the configuration of the upper aluminium alloy sheet. On the other hand, the three sheets for the configuration of the lower aluminium alloy sheet were not joined even by optimizing the die shape in the both finite element simulation and experiment, because the material flow of the steel sheets was insufficient to form the two interlocks. The tension-shear loads for the clinched and riveted sheets with the adhesive were almost the same, because the load for the adhesive was the highest. In the cross-tension test, however, the load by the adhesive was comparatively small.

## 1. Introduction

The use of multi-materials in the automobile body has been increasing to reduce the weight of automobiles, and steel sheets, aluminium alloy sheets and carbon fiber reinforced plastics are used for automobile body parts. Sheet combinations that include these various materials are not easy to join by conventional resistance spot welding for lapped steel sheets. Instead of the conventional welding process, various joining methods such as friction stir welding, joining by flow drilling screw, high-speed bolt joining, self-pierce riveting, mechanical clinching and adhesive bonding have been developed and used [1,2]. Although joining two sheets of multi-materials is usually done, not only joining two sheets, but also joining three or more sheets is attractive when creating an automobile body. Joining by the flow drilling screw and high speed bolt joining may be possible to join three or more sheets because all sheets are driven through by the flow drilling screw and the bolt, respectively. However, for the corrosion resistance and the seal in joints, the joining processes without fracture in a sheet or more sheets such as friction stir welding, self-pierce riveting, mechanical clinching and adhesive bonding are attractive. In the case of joining one aluminium alloy sheet and two steel sheets, friction stir welding is difficult because a tool strength is not enough to stir the steel sheet. Although adhesive bonding is possible to join the sheet configuration, the curing time of an epoxy resin is not sufficiently short to assemble automobile parts in the manufacturing process. Therefore, self-pierce riveting, mechanical clinching and these joining processes with an adhesive are the alternative methods to join the sheet configuration for one aluminium alloy sheet and two steel sheets.

Self-pierce riveting is a cold process for joining sheets by driving a rivet through the upper sheet and by flaring a skirt of the rivet to create an interlock between the skirt and the lower sheet. By hooking the lower sheet on the flared skirt underneath and embedding the rivet in the upper sheet, the sheets are joined. He et al. [3] reviewed self-pierce riveting processes in 2008. The self-pierce riveting process is suitable to join the aluminium alloy parts in the automobiles [4]. To join aluminium alloy sheets for automobile parts, the riveting process had been simulated numerically using the finite element code LS-DYNA as a two-dimensional axisymmetric model including the two aluminium alloy sheets to be joined, rivets and tools [5]. The simulation of self-pierce riveting of two 6000 series aluminium alloy sheets was compared with experimental results [6]. In a finite element model of the self-pierce riveting process for aluminium alloy sheets, the equations that govern the onset and propagation of cracks during the self-pierce riveting process were considered [7]. Although the conventional rivets are made of boron steel, aluminium rivets were developed for joining 5000 [8] and 6000 [9] series aluminium alloy sheets, respectively. The effect of the rivet coatings on the strength of riveted aluminium alloy sheets was investigated [10]. The joint distortion in self-pierce riveted 5000 series aluminium alloy sheets was investigated, and the distortion was much larger than that in the resistance spot welded sheets [11]. The behaviors of the self-pierce riveted 6000 series aluminium alloy joints under quasi-static loading conditions were investigated by the experiment and the simulation [12]. Three-dimensional simulation of self-pierce riveting of the two aluminium alloy sheets was performed to calculate the shear strength of the joined sheets [13]. The joint performance of self-pierce riveted 5000 series aluminium alloy sheets at automotive crash speeds was investigated [14]. As shown in these works above, the riveting processes and the properties of joints for the aluminium alloy sheets have been developed and investigated. The development of riveting processes has been expanded to join the aluminium alloy and steel sheets. The joinability of aluminium alloys and mild steel sheets using a self-piercing rivet was evaluated by using a finite element simulation and experiment [15]. The effect of coatings on the quality and the behavior of self-pierce riveted joints between 5000 series aluminium alloy and high strength steel sheets was investigated [16]. The effects of the rivet hardness, the rivet length and the die shape on the deformation behaviors and the mechanical performance of 6000 series aluminium alloy and mild steel sheets were investigated [17]. In self-pierce riveting for the two sheets of high strength steel and aluminium alloy sheets, the effect of the steel strength on the joinability was evaluated [18]. An ultra-high strength steel sheet was joined with an aluminium alloy sheet by self-pierce riveting [19]. To join not only the high strength steel and aluminium alloy sheets, but also both the high strength steel sheets, the rivet geometry for the self-pierce riveting was improved [20]. Not only joinability, but also the strength of the joined sheets was evaluated. The numerical modelling of the self-pierce riveting process to the final assembled parts was developed, the mechanical strength for the assembled aluminium alloy and the steel parts was predicted [21]. The strength of the joined 5000 series aluminium alloy and high strength steel sheets by self-pierce riveting and welding was compared [22]. The dynamic strength of joined 5000 series aluminium alloys and high strength steel sheets by self-pierce riveting was evaluated [23]. Although two sheets are joined in these works above, it is desirable to join three sheets for improving the structure design in the automobile body. The effect of sheet configurations in the three sheets including the aluminium alloy and high steel sheets on the deforming behaviors was investigated [24]. The three sheets including the high strength steel and the aluminium alloy sheets in the thick lower sheet were joined by self-pierce riveting [25]. Although the steel sheet including the 980 MPa ultra-high strength steel sheet was joined in self-pierce riveting of the three sheets, the limit ratio of the lower sheet thickness was about 0.4 in the total thickness, i.e., the ratio is not small and it was shown that the small ratio of the lower sheet thickness is difficult to join [26].

Mechanical clinching is a cold joining process of sheets by local hemming with a punch and die without a screw or a rivet. The recent developments in the improved clinching processes have been reviewed [27]. The production cost of mechanical clinching with that of the self-pierce riveting was compared [28], and mechanical clinching is useful for joining automobile body panels because of low running costs. By mechanical clinching, the joining processes for two sheets of similar materials such as aluminium alloy sheets [29,30], steel sheets [31,32] and die-quenched steel sheets [33,34] have been developed. The joining processes for dissimilar materials such an aluminium alloy sheet and mild steel sheet [35], a mild steel sheet and aluminium casting [36], an aluminium alloy sheet and high strength steel sheet [37], an aluminium alloy sheet and ultra-high strength steel sheet [38], an aluminium alloy sheet and die-quenched steel sheet [39,40] and a carbon fiber-reinforced plastic and aluminium alloy sheet [41,42] have been developed. Although the mechanical clinching processes in these works may involve additional processes such as heating or the use of a pre-punched hole, it becomes possible to join many sheet combinations in the automobile body. Not only two sheets, but also three sheets are joined by mechanical clinching. Kaðèák et al. have joined the three steel sheets [43] and a sheet combination including the high strength steel and aluminium alloy sheets [44]. However, the three sheets including the ultra-high strength and aluminium alloy sheets are not tried.

**Figure 1:**
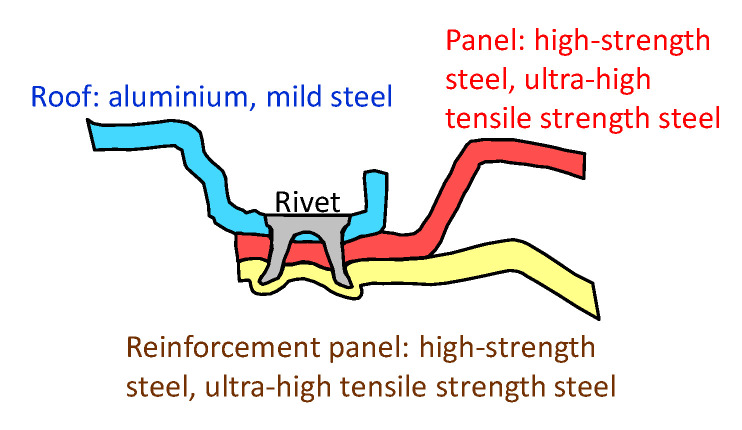
Self-pierce riveting of three sheets in automobile body parts.

In the case of the automobile, body parts like the sheet configuration of the roof panel, the panel and the reinforcement panel, three sheets of one thin aluminium alloy sheet and two thin ultra-high strength steel sheets are attractive for lightweight in Figure 1, although the welded three steel sheets are conventionally used. It is not easy to join this sheet configuration including one thin soft aluminium alloy sheet and two thin hard steel sheets by self-pierce riveting and mechanical clinching because of the small thickness ratio, the high flow stress with the low ductility of steel and the large difference of flow stresses.

In this study, three sheets of one thin 5000 series aluminium alloy sheet and two thin 980 MPa ultra-high strength steel sheets were joined by self-pierce riveting and mechanical clinching. The joinabilities for two kinds of sheet configurations in the both processes were investigated with different die shapes. Then the joint strengths with and without adhesive were compared.

## 2. Self-Pierce Riveting 

### 2.1. Self-Pierce Riveting Process of Three Sheets

**Figure 2:**
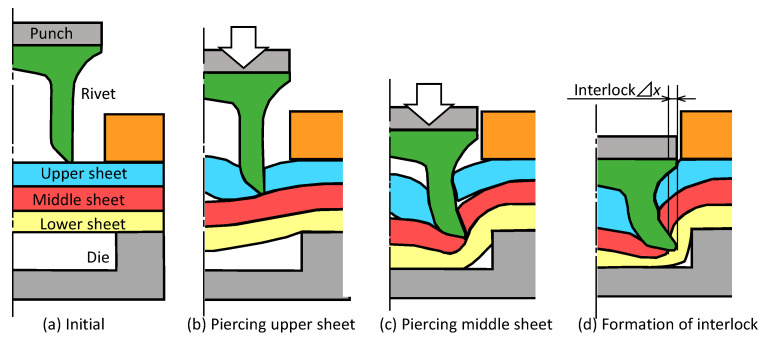
Self-pierce riveting process of three sheets: (**a**) initial; (**b**) piercing upper sheet; (**c**) piercing middle sheet; (**d**) formation of interlock.

The self-pierce riveting process of three sheets is shown in Figure 2. The self-pierce rivet is driven through the sheets except for the lowest sheet and the rivet skirt is flared in the lowest sheet. The sheets are mechanically joined by the interlock between the lower sheet and the flared skirt of the rivet. Joining three sheets in the self-pierce riveting process requires:

- driving a rivet skirt through all sheets except for the lowest sheet;
- interlock formation by flaring rivet skirt in the lowest sheet;
- no fracturing of the lowest sheet.

When the rivet skirt is not driven though the sheets except for the lowest sheet, the sheets do not join, i.e., the interlock in the lowest sheet is not formed. The fracture in the lowest sheet brings corrosion.

**Figure 3:**
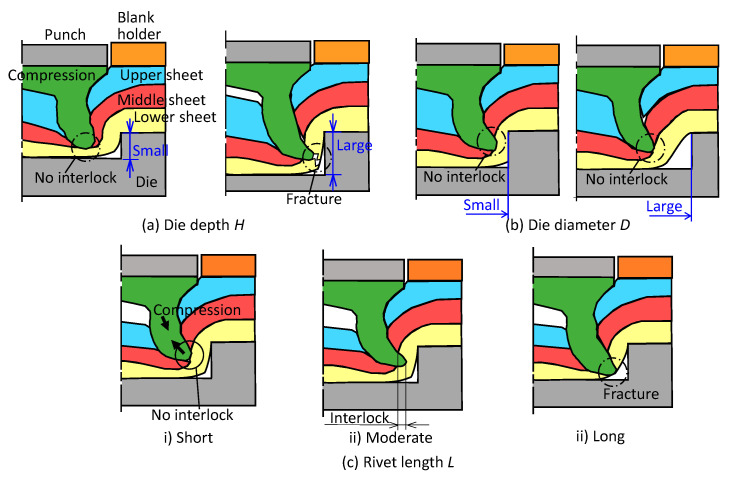
Effects of die shape and rivet length on the deforming behavior of sheets and rivets in the self-pierce riveting of three sheets: (**a**) die depth *H*; (**b**) die diameter *D*; (**c**) rivet length *L*.

The effects of the die shape and the rivet length on the deforming behavior of sheets and rivets in self-pierce riveting of three sheets are shown in Figure 3. In the small die depth in Figure 3a, the interlock does not form due to insufficient deformation. On the other hand, a fracture may occur in the excessive die depth due to the large deformation of the lower sheet. In both the too small and the excessive diameter of die in Figure 3b, the interlock does not form. The interlock without the fracture forms in the moderate depth and diameter of the die. During penetrating the rivet through the upper and middle sheets, the rivet length tends to be shortened by the compressive stress. In this case, the interlock may not be formed due to the insufficient length in Figure 3c. Thus the interlock is formed by the moderate rivet length, whereas the fracture occurs in the excessive length. In this study, two kinds of the sheet configurations were carried out, and thus the one is one aluminium alloy-two 980 MPa steel sheets, and the other is two 980 MPa steel-one aluminium alloy sheets. Although the deformation behavior of the sheets is controlled by the die shape, the behavior depends on the sheet configuration. In the upper aluminium alloy sheet, not only the penetrating of the rivet through the hard middle ultra-high strength steel sheet, but also the flaring skirt of the rivet without fracture is difficult due to such a hard material with a low ductility. In the lower aluminium alloy sheet, penetrating the rivet through the hard upper and middle ultra-high strength steel sheets is the main problem.

### 2.2. Sheet Materials

One 5000 series aluminium alloy sheet and two 980 MPa steel sheets as ultra-high strength steel sheets for automobile body panels are used. The ultra-high strength steel is a dual phase steel. The thickness of these sheets is 1 mm. The material properties of the sheets are shown in Table 1. The properties were obtained by a uni-axial tension test. The testing machine was a 50 kN screw driven type universal testing instrument (Autograph AGS-J, SHIMADZU Co., Kyoto, Japan). The three tensile specimens were pulled, and the averages are shown.

As an example in Figure 1, an aluminium alloy sheet is the roof panel, and two ultra-high strength steel sheets are the panel and the reinforcement panels, respectively. In stacking these sheets for joining, a configuration of the aluminium alloy-steel-steel sheets is usually selected. The opposite stacking, and thus the other configuration of the steel-steel-aluminium alloy sheets is also an alternative selection.

### 2.3. Experimental and Numerical Conditions of Self-Pierce Riveting

**Figure 4:**
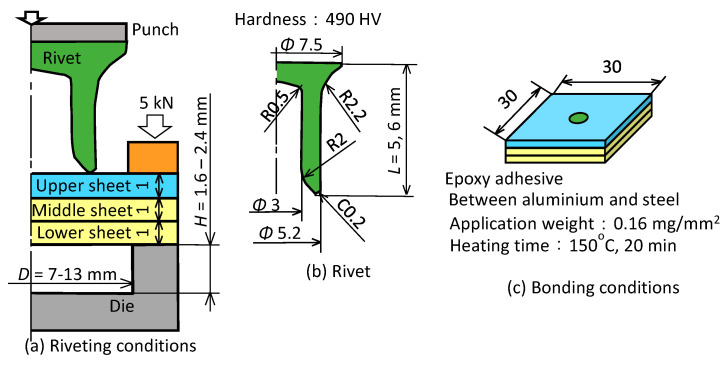
Self-pierce riveting conditions of three sheets: (**a**) riveting conditions; (**b**) rivet; (**c**) bonding conditions.

The self-pierce riveting conditions of three sheets are shown in Figure 4. The three sheets were fixed with the sheetholder and die, and were joined by driving the rivet with the punch. The rivet was pressed by the punch until the top surface of the rivet was located at the same height as the upper surface of the upper sheet, whereas the interlock may increase with the increased stroke. The press was the 800 kN CNC servo press (SDE-8018, AMADA MACHINERY Co., Kanagawa, Japan). The effect of the die shape was investigated. Although the rivet of 5 mm in length was usually used for the two sheets, the longer rivet was used to pierce the upper and middle sheets. The rivet is made of boron steel (490 HV) and is plated with a zinc alloy to prevent corrosion. The sheet was 30 mm in length on its side. The ratio of lower sheet thickness and total thickness is 0.33. Because the riveted and clinched joints sometimes used an adhesive in the actual joining process, riveting and clinching with the epoxy adhesive (EP138, CEMEDINE Co., Tokyo, Japan) were also performed. The static strength of the clinched and self-piercing riveted joints with the adhesive was essentially due to the adhesive layer, and the high joint strength was sometimes given by bonding [45]. The adhesive was only applied between aluminium and steel sheets, and the application weight was 0.16 mg/mm^2^. To harden the adhesive after joining, the sheets with the adhesive were heated at 150 °C for 20 min in an electric furnace. The lengths of the interlock and the minimum wall thickness were measured from the cut joined sheets by a digital microscope (VHX-1000, KEYENCE Co., Osaka, Japan).

**Figure 5:**
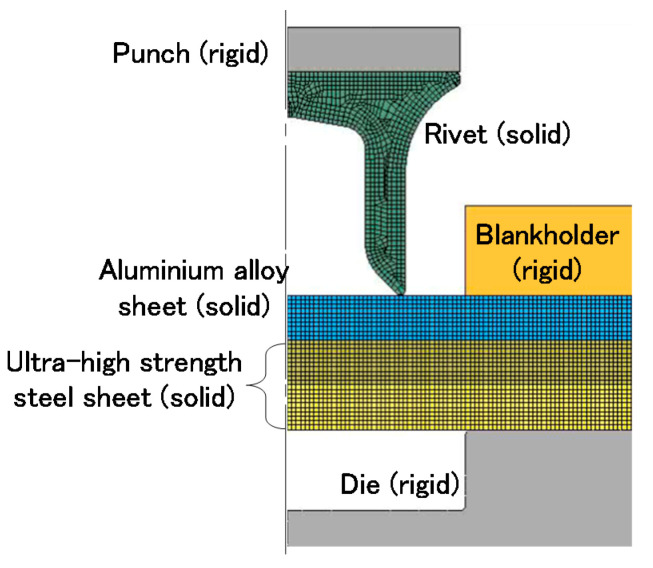
Conditions of simulation in self-pierce riveting for three sheets and for the upper aluminium alloy sheet.

The conditions of the simulation in self-pierce riveting for three sheets are shown in Figure 5. The self-pierce riveting process of the three sheets was simulated by the commercial finite element code LS-DYNA and JVISION (JSOL Corp., Tokyo, Japan). By limiting the calculation to the vicinity undergoing plastic deformation, axi-symmetric deformation was assumed. Since the sheets and rivet undergo plastic deformation during the riveting, the cross-sections of the rivet and sheets were divided into quadrilateral solid elements with 0.1 mm × 0.1 mm in the mesh size of an element. In the simulation, adaptive remeshing of elements was automatically performed when the distortion of the element was large. The die, the punch and the sheetholder were assumed to be rigid. The Coulomb friction was assumed at the interfaces and the coefficient of friction at the interfaces equivalent to an unlubricated condition in the simulation was 0.20. The flow stress curves of the sheets measured from the tensile test were used for the finite element simulation. In the simulation, riveting without the adhesive was only performed, although the effects of mechanical joining and adhesive bonding have been tried numerically and experimentally [46].

### 2.4. Upper Aluminium Alloy Sheet

**Figure 6:**
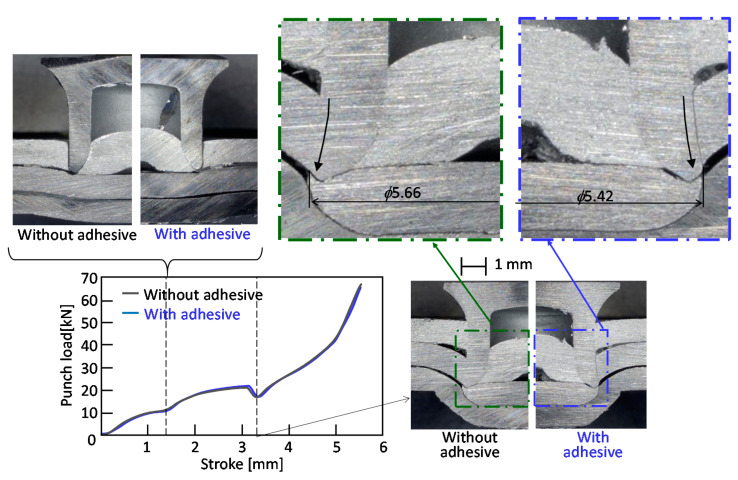
Deforming behaviors of sheets and rivet and punch load-stroke curves for *D* = 8 mm, *H* = 1.8 mm and *L* = 5 mm obtained from the experiment.

The deforming behaviors of the sheets and rivet and punch load-stroke curves for *D* = 8 mm, *H* = 1.8 mm and *L* = 5 mm obtained from the experiment are shown in Figure 6. Although both punch the load-stroke curves are same, the deforming behaviors of the upper sheet with and without the adhesive are slightly different. It seems that flaring of the rivet with the adhesive is a little smaller by the adhesive.

**Figure 7:**
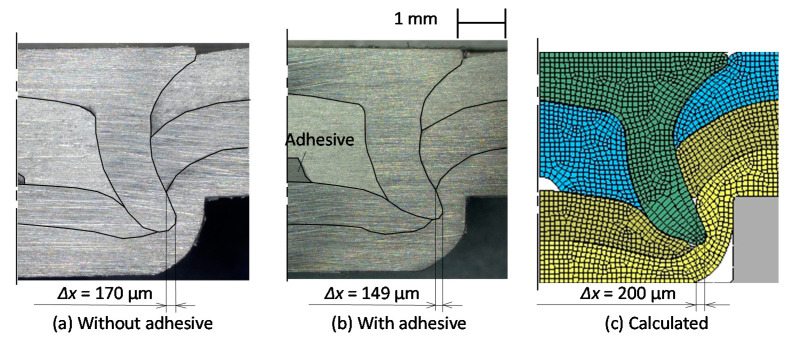
Cross-sectional shapes of the rivet and sheets obtained from experiment and calculation for *D* = 8 mm, *H* = 1.8 mm and *L* = 5 mm: (**a**) without adhesive; (**b**) with adhesive; (**c**) calculated.

The cross-sectional shapes of the rivet and sheets obtained from the experiment and the calculation are illustrated in Figure 7. The interlock with the adhesive in Figure 7a is smaller than that without the adhesive in Figure 7b because of small flaring of the rivet in Figure 6. The adhesive remains at the center between the upper and middle sheets in Figure 7b, whereas the gaps at the center between the upper and middle sheets without the adhesive in both the experiment and calculation are smaller. The calculated shape shown in Figure 7c is similar to the experimental one in Figure 7a, although the calculated interlock is larger than the experimental one.

**Figure 8:**
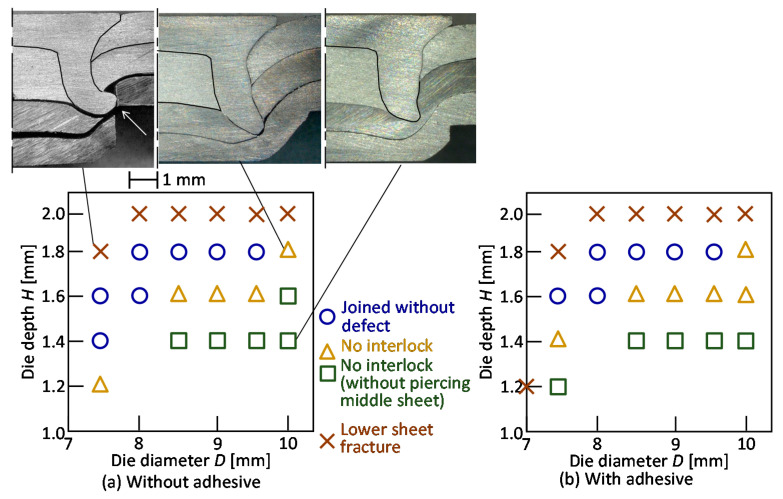
Joining ranges for *L* = 5 mm: (**a**) without adhesive; (**b**) with adhesive.

The joining ranges for *L* = 5 mm are shown in Figure 8. As a defect, the no interlock with and without piercing middle sheet and the lower sheet caused a fracture. An interlock less than 50 micrometers was classified to the no interlock. In the smaller and die depth, no interlock and lower sheet fractures tended to occur, respectively. This tendency with the adhesive is same with that without the adhesive, and the range is little smaller than that without the adhesive.

**Figure 9:**
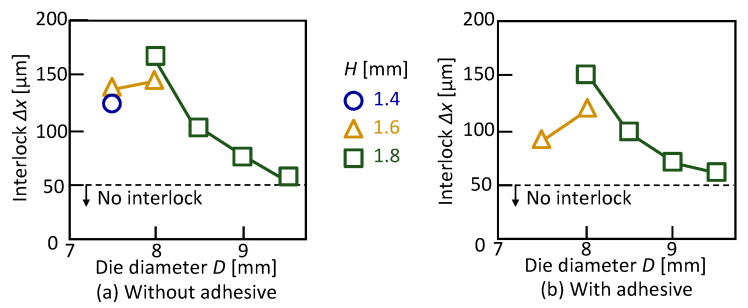
Effect of die shape on the interlock for *L* = 5 mm: (**a**) without adhesive; (**b**) with adhesive.

The effect of the die shape on the interlock for *L* = 5 mm is shown in Figure 9. The interlock has a peak for the die diameter, the maximum interlock was obtained in *D* = 8.0 mm and *H* = 1.8 mm without the adhesive. Although this tendency and the optimum condition with the adhesive are the same as that without the adhesive, the maximum interlock is smaller than that without the adhesive as shown in Figure 7. In the sheet configuration of one aluminium alloy-two 980 MPa steel sheets, the three sheets were successfully joined by self-pierce riveting, and the optimum conditions are *D* = 8 mm, *H* = 1.8 mm and *L* = 5 mm both with and without the adhesive.

### 2.5. Lower Aluminium Alloy Sheet

**Figure 10:**
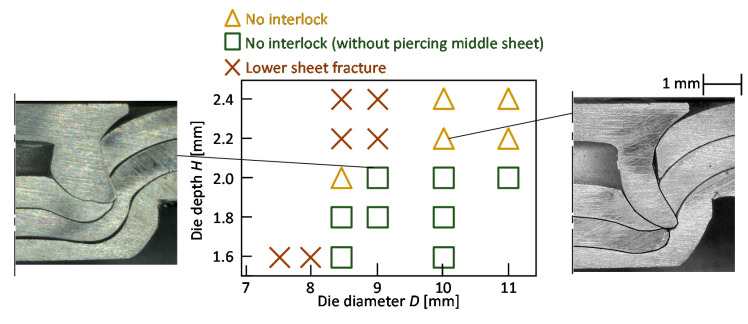
Joining range for *L* = 5 mm and without adhesive.

The joining range for *L* = 5 mm and without the adhesive is shown in Figure 10. The sheets are not joined without the defects. In some conditions, a very small interlock less than 50 micrometers was observed.

**Figure 11:**
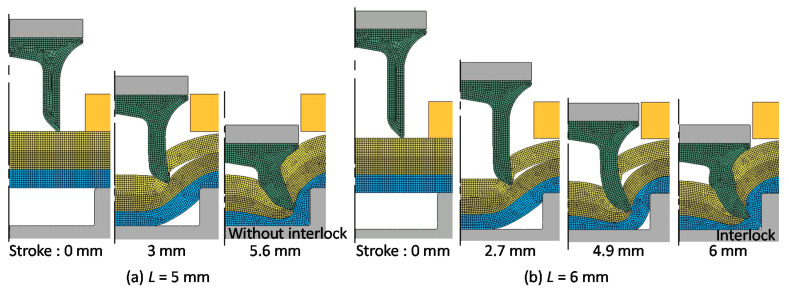
Calculated deforming behaviors of sheets and rivet for *D* = 9 mm and *H* = 2 mm, (**a**) *L* = 5 mm; (**b**) *L* = 6 mm.

To increase the interlock as shown in Figure 3c, a long rivet was used. The calculated deforming behaviors of the sheets and rivet for *D* = 9 mm and *H* = 2 mm were shown in Figure 11. Although the rivet does not pierce through the middle sheet in *L* = 5 mm because of the high strength of the upper and middle sheets, the interlock is successfully formed by the long rivet for *L* = 6 mm.

**Figure 12:**
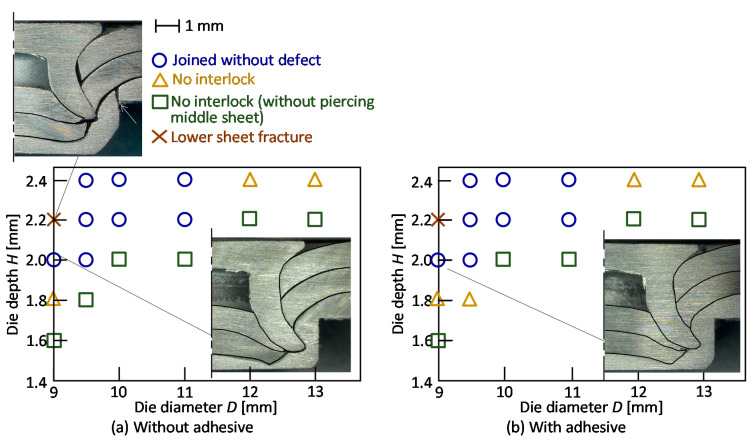
Joining ranges (**a**) without and (**b**) with adhesive for *L* = 6 mm.

The joining ranges with and without the adhesive for *L* = 6 mm are shown in Figure 12. In the small diameter and the large depth of the die, the sheets are joined without defects. This tendency with the adhesive is the same as that with as that without the adhesive, and the range is similar to the range without the adhesive.

**Figure 13:**
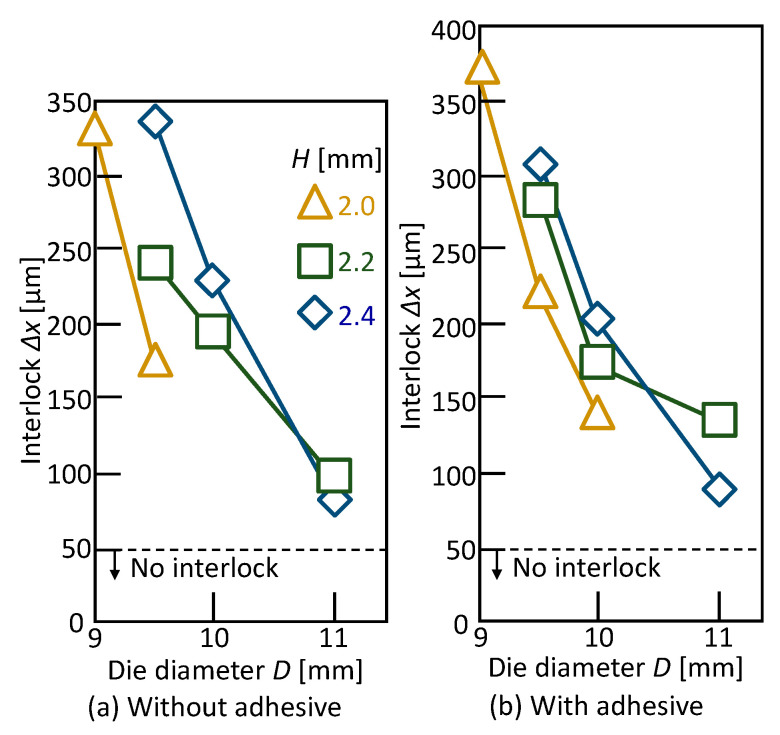
Effect of the die shape on interlock for *L* = 6 mm: (**a**) without adhesive; (**b**) with adhesive.

The effect of the die shape on the interlock for *L* = 6 mm is shown in Figure 13. The interlock decreases with an increasing die diameter. As the optimum conditions, *D* = 9 mm, *H* = 2 mm and *L* = 6 mm both with and without the adhesive are selected. In the sheet configuration of two 980 MPa steel-one aluminium alloy sheets, the sheets were successfully joined by self-pierce riveting using the long rivet.

## 3. Mechanical Clinching

### 3.1. Mechanical Clinching Process of Three Sheets

**Figure 14:**
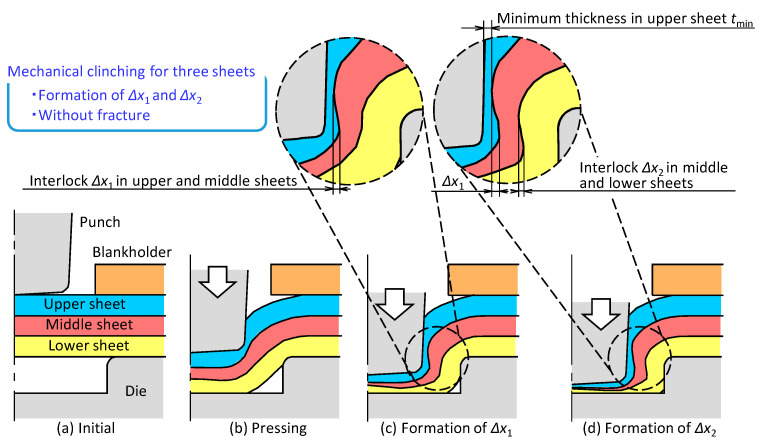
Mechanical clinching process of three sheets: (**a**) initial; (**b**) pressing; (**c**) formation of *Δ*x_1_; (**d**) formation of *Δ*x_2_.

The mechanical clinching process of three sheets is shown in Figure 14. The sheets are joined by the two interlocks, i.e., one is formed between the upper and middle sheets, and the other is formed between the middle and lower sheets. Joining three sheets in mechanical clinching requires:

- interlock formation between upper and middle sheets;
- interlock formation between middle and lower sheets;
- no fracturing of sheets.

To obtain the joint strength, not only formation of the two interlocks, but also the certain minimum wall thickness *t*_min_ is required.

**Figure 15:**
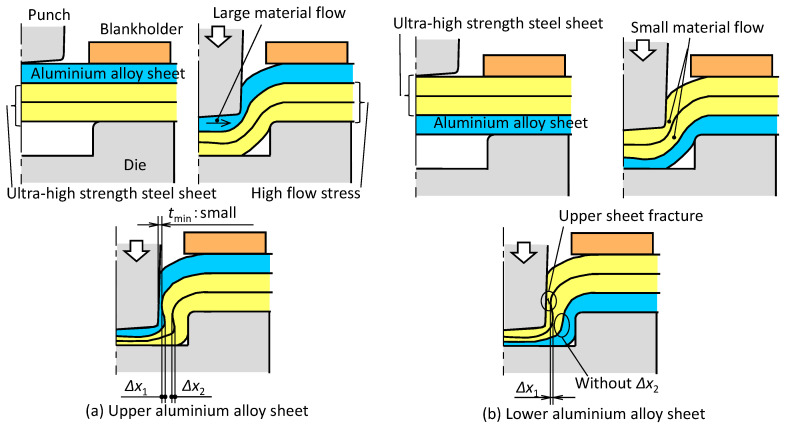
Effects of sheet combinations on the deforming behavior of sheets: (**a**) upper aluminium alloy sheet; (**b**) lower aluminium alloy sheet.

The effects of the sheet configurations on the deforming behavior of the sheets are shown in Figure 15. In the upper aluminium alloy sheet, the material flow of the soft upper sheet is large because of the high flow stress of the middle and lower ultra-high strength steel sheet, and both the minimum wall thickness and the two interlocks become small. In the lower aluminium alloy sheet, the material flows of the upper and middle sheets become small and limited due to the high flow stress and low ductility, i.e., the interlocks are not formed, and the upper sheet tends to have fracture.

**Figure 16:**
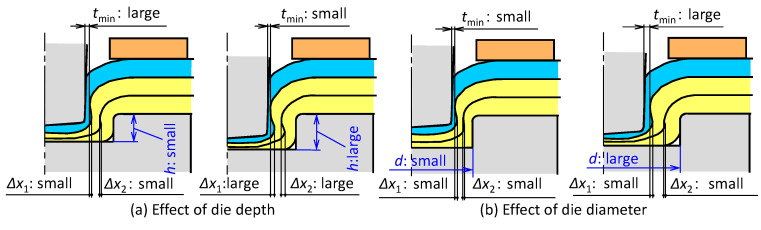
Effects of die shape on the deforming behavior of sheets: (**a**) die depth; (**b**) die diameter.

The effects of the die shape on the deforming behavior of the sheets are shown in Figure 16. The deforming behaviors of the sheets are controlled by the die depth *h* and the die diameter *d*. The small die depth brings small deformation, and thus the minimum wall thickness of the upper sheet becomes large, and the interlocks are small. In the large depth, the large interlocks are obtained, whereas the large deformation of the upper sheet is brought. In the small diameter of die, all of the minimum wall thicknesses of the upper sheet and interlocks become small. Although the interlocks increase with an increasing die diameter, the interlocks decrease in the excessive die diameter due to the large material flow to the outside in the die cavity.

### 3.2. Conditions of Mechanical Clinching

**Figure 17:**
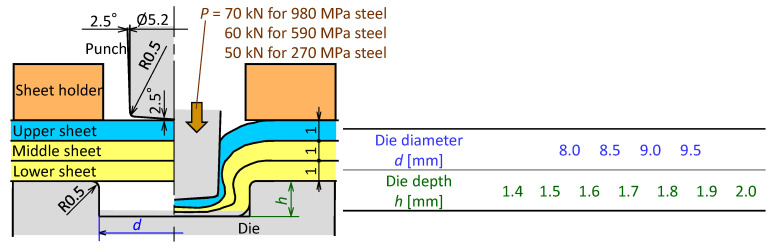
Mechanical clinching conditions of three sheets.

The mechanical clinching conditions of three sheets are shown in Figure 17. The three sheets were fixed with the sheet holder and die, and were joined by the punch pressed. The effect of the die shape was investigated. To compare the results, not only the 980 MPa ultra-high strength steel sheets, but also the 590 MPa grade high strength and 270 MPa grade strength steel sheets were used. To increase the interlocks, the punch load increased for the higher strength of steel sheets. However, the punch force was limited to a maximum of 70 kN for 980 MPa steel sheets to prevent buckling of the tool steel punch. Mechanical clinching with the adhesive was also performed and the same joining conditions used in Figure 4.

### 3.3. Upper Aluminium Alloy Sheet

**Figure 18:**
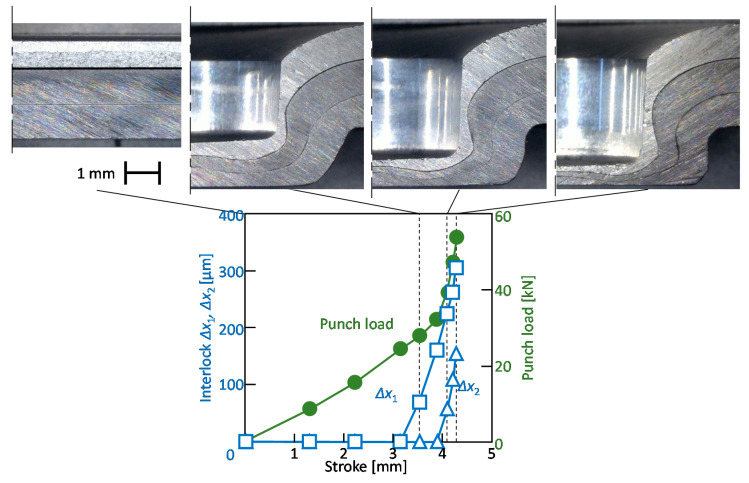
Deforming behaviors of sheets and punch load-stroke curves for *d* = 9.0 mm, *h* = 1.7 mm, without adhesive and 270 MPa steel sheets obtained from the experiment.

The deforming behaviors of the sheets and punch load-stroke curves for *d* = 9.0 mm, *h* = 1.7 mm and the 270 MPa steel sheets obtained from the experiment are shown in Figure 18. The punch load increases with an increase in the punch stroke. The formation of the interlock between the upper and middle sheets starts at 3.1 mm in stroke, and the formation of the interlock between the middle and lower sheets starts at 3.8 mm in stroke. Both the interlocks are formed without fracture, and the three sheets are joined by mechanical clinching.

**Figure 19:**
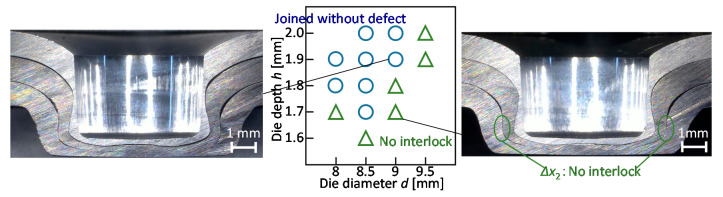
Joining range for 980 MPa steel sheets and without adhesive.

The joining range for the 980 MPa steel sheets and without the adhesive is shown in Figure 19. In the large depth and small diameter of the die, the three sheets are joined without fracture.

**Figure 20:**
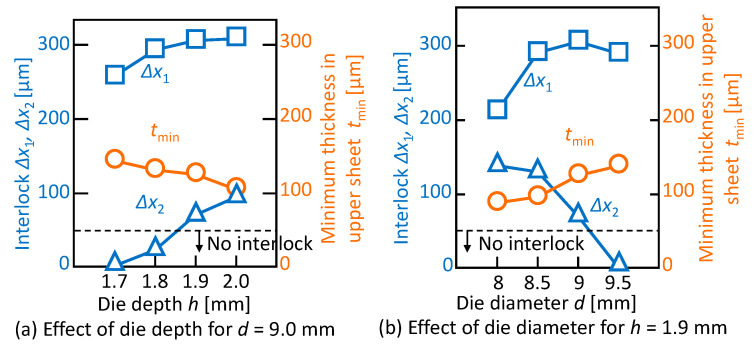
Effect of die shape on interlocks and minimum thickness in the upper sheet: (**a**) die depth for *d* = 9.0 mm; (**b**) die diameter for *h* = 1.9 mm.

The effect of the die shape on the interlocks and the minimum thickness in the upper sheet is shown in Figure 20. In *d* = 9 mm, the minimum thickness decreases and the interlocks between the upper and middle sheets increase with an increasing die depth. As the die diameter increases in *h* = 1.9 mm, the minimum thickness and the interlock between the upper and middle sheets increase, whereas the interlock between the middle and lower sheets decreases. In the sheet configuration of one aluminium alloy-two 980 MPa steel sheets, the three sheets are successfully joined by mechanical clinching, and the conditions of *d* = 9 mm and *h* = 1.9 mm are selected as the optimum conditions.

**Figure 21:**
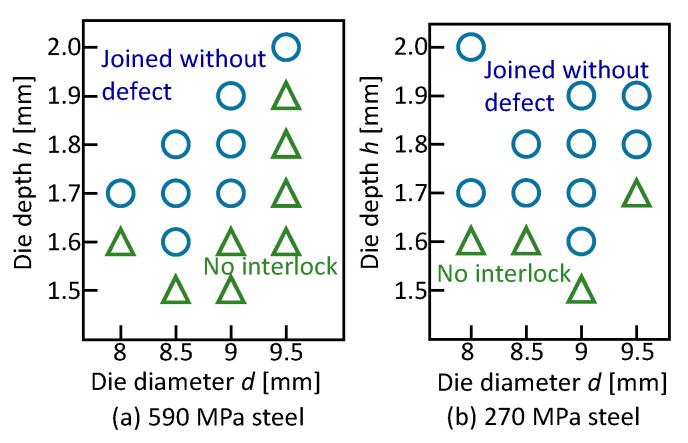
Joining ranges for (**a**) 590 MPa and (**b**) 270 MPa steel sheets and without adhesive.

The joining ranges for the 590 MPa and 270 MPa steel sheets and without the adhesive are shown in Figure 21. Although the range for the 590 MPa steel is similar to the range for the 980 MPa steel, the range for the soft 270 MPa steel is wider.

**Figure 22:**
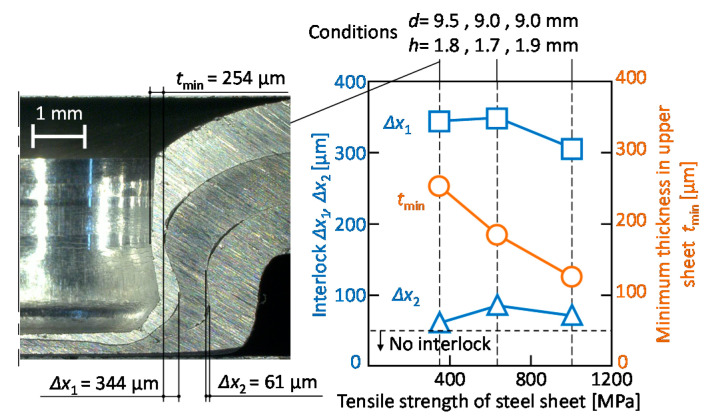
Effect of tensile strength of steel sheet on the interlocks and the minimum thickness in upper sheet.

The effect of the tensile strength of steel sheets on the interlocks and the minimum thickness in the upper sheet is shown in Figure 22. To obtain the high joint strength for the 590 MPa and 270 MPa steel, the die shape obtained at the maximum minimum thickness in the upper sheet was shown. Although the effect of the tensile strength of the steel sheet on the interlocks is small, the minimum thickness in the upper sheet decreases with an increase in the tensile strength of the steel sheet because the deformation of the aluminium alloy sheet increases.

### 3.4. Lower Aluminium Alloy Sheet

**Figure 23:**
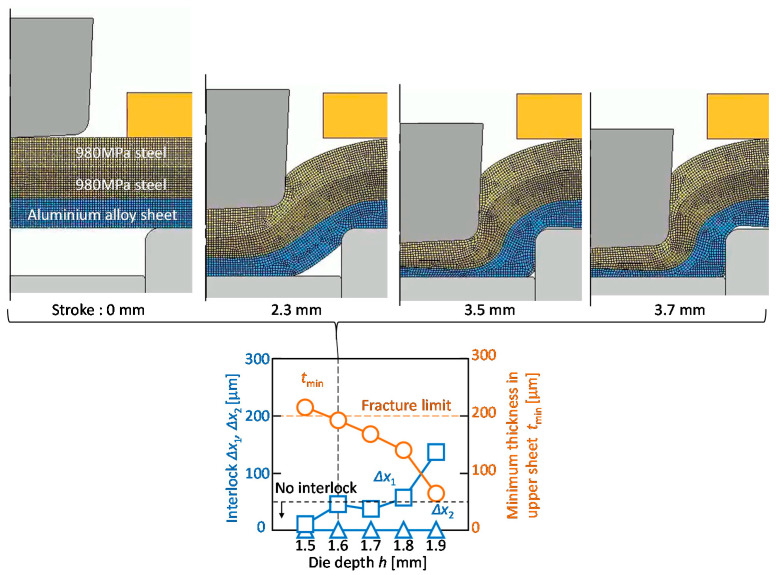
Effect of die depth on calculated interlocks and minimum thickness in upper sheet for *d* = 9 mm and 980 MPa steel sheets.

The effect of the die depth on the calculated interlocks and the minimum thickness in the upper sheet for *d* = 9 mm and 980 MPa steel sheets are shown in Figure 23. In the graph, the fracture limit for upper sheet in the experiment is added. The minimum thickness in the upper sheet decreases with an increasing of the die depth. The calculated minimum thickness reaches the limit at *h* = 1.6 mm. Although the interlock between the upper and middle sheets increases with increasing of the die depth, the interlock between the middle and lower sheets does not form because of the insufficient material flow of the steel sheets. It seems that the sheet configuration of two 980 MPa steel and one aluminium alloy sheets is hard to join by mechanical clinching.

**Figure 24:**
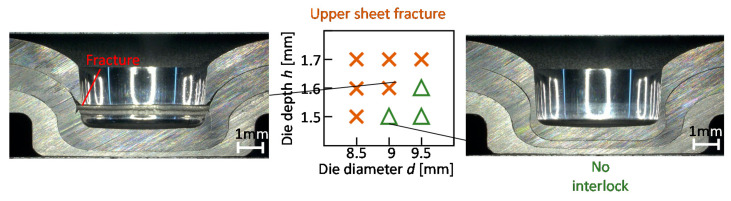
Joining range for sheet combination of two 980 MPa steel and aluminium alloy sheets.

The joining range for the sheet configuration of two 980 MPa steel and one aluminium alloy sheets was investigated by the experiment. The joining range is shown in Figure 24. In the large depth and small diameter of die, the fracture in the upper sheet occurs because of the low ductility of the upper sheet. The interlocks are not formed by the small depth and the large diameter of the die to decrease the deformation of the upper sheet for the prevention of fracture. Thus, the sheet configuration of two 980 MPa steel sheets and one aluminium alloy sheet is difficult to join by the modification of die shape in mechanical clinching.

**Figure 25:**
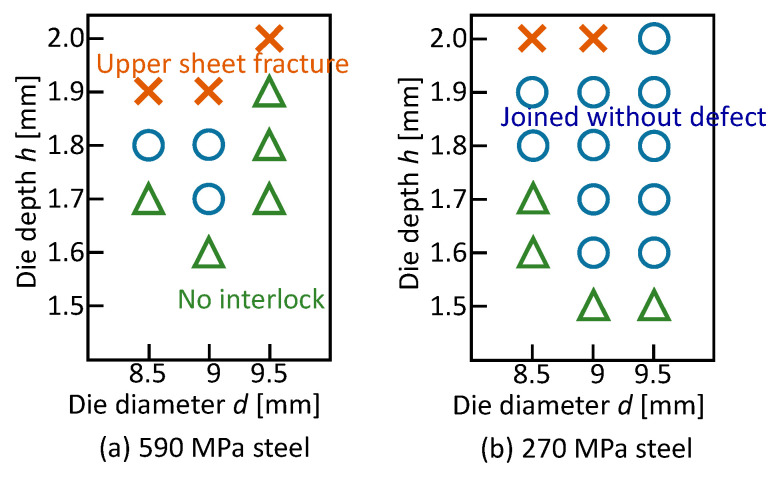
Joining ranges for (**a**) 590 MPa and (**b**) 270 MPa steel sheets.

Instead of the 980 MPa steel sheets, the 590 MPa and 270 MPa steel sheets were used, and the joining ranges are shown in Figure 25. In the excessive die depth, the upper sheet fracture occurs and the interlock does not form in the too-small die depth. In both the sheet configurations, three sheets are joined without defects in some conditions. The joining range for the 270 MPa steel with the low flow stress and high ductility is wider.

**Figure 26:**
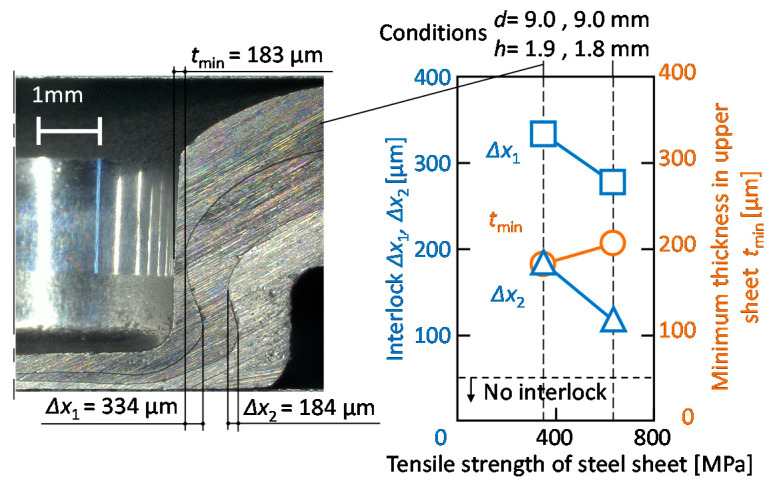
Effect of the tensile strength of the steel sheet on the interlocks and the minimum thickness in the upper sheet.

The effect of the tensile strength of steel sheet on the interlocks and the minimum thickness in the upper sheet is shown in Figure 26. To obtain the high joint strength for the 590 MPa and 270 MPa steel, the die shape obtained from the maximum minimum interlock between the middle and lower sheet was shown. Although the minimum thickness in the upper sheet is slightly increased, the interlocks are decreased with an increase in the tensile strength of the steel sheet.

## 4. Joint Strength

### 4.1. Measuring Conditions of Joint Strength

**Figure 27:**
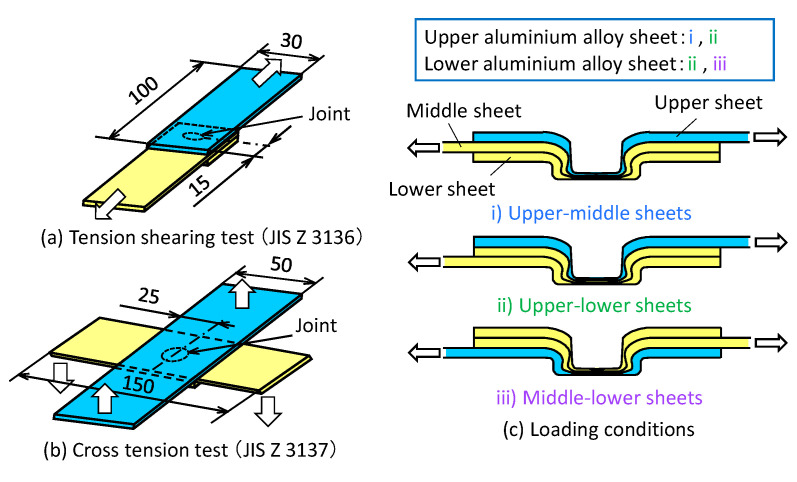
Measuring conditions of joint strength: (**a**) tension-shearing test (JIS Z 3136); (**b**) cross-tension test (JIS Z 3137); (**c**) loading conditions.

The measuring conditions of the joint strength are shown in Figure 27. The tension-shearing and the cross-tension tests were performed based on the test for the resistance spot welded joints [47,48]. The three sheets were joined by self-pierce riveting and mechanical clinching with the optimum condition show in Section 2 and Section 3. The aluminium alloy sheet was pulled under constraining the other steel sheet, i.e., in the upper aluminium alloy sheet, the tests in the upper–middle sheets and the upper-lower sheets were performed. In the test, a 50 kN screw driven type universal testing instrument (Autograph AG-IS, SHIMADZU Co., Kyoto, Japan) was used, and the tensile speed was 10 mm/min.

### 4.2. Upper Aluminium Alloy Sheet

**Figure 28:**
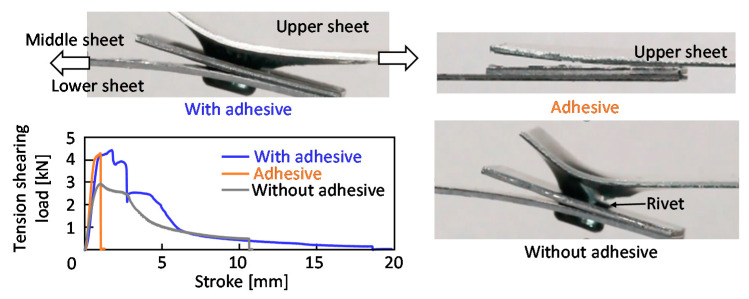
Tension-shearing load and stroke curves of upper-lower sheets for self-pierce riveted sheets, *D* = 8 mm, *H* = 1.8 mm and *L* = 5 mm.

The tension-shearing load and the stroke curves of the upper-lower sheets for the self-pierce riveted sheets, *D* = 8 mm, *H* = 1.8 mm and *L* = 5 mm, are shown in Figure 28. As a comparison, the curve of the adhesive joint without riveting is added in the figure. The maximum loads of the adhesive joint and the riveted joint with the adhesive are similar, and are higher than that of the riveted joint without the adhesive. The strokes of the riveted joint with and without the adhesive are longer than the adhesive joint, and thus the absorbed energy of the joint including riveting is higher than that of the adhesive joint.

**Figure 29:**
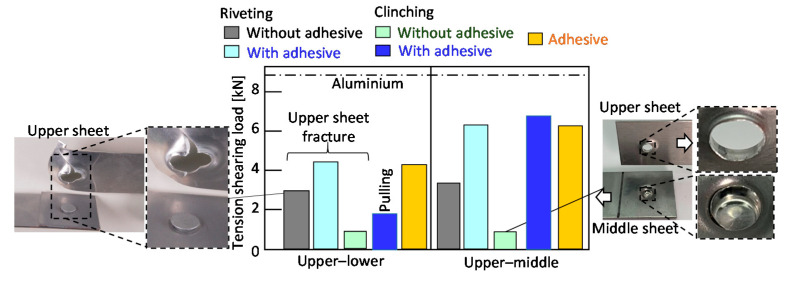
Tension-shearing loads of riveted and clinched joints for the upper aluminium alloy sheet.

The tension-shearing loads of the joints are summarized in Figure 29. The tension-shearing load of the aluminium alloy sheet calculated from the tensile strength and the cross-sectional area is about 8.7 kN, and is added in the figure. In the upper–lower sheets, the loads of the adhesive joint and the riveted joint with the adhesive are the highest because of the high strength of the adhesive. Although the load of the clinched joint with the adhesive is increased by the adhesive, the increment is small because the fracture occurred between the middle and lower sheets without the adhesive. In the upper–middle sheets, the load of the riveted joint without the adhesive is similar to the load in the upper–lower sheets due to the low strength of the upper sheet. The tendency is the same in the load of the clinched joint without the adhesive. The loads of the riveted and clinched joints with the adhesive are similar, and are higher than the joints without the adhesive. Thus, the clinched joint with the adhesive for improving the shear joint strength is useful without the rivet cost.

**Figure 30:**
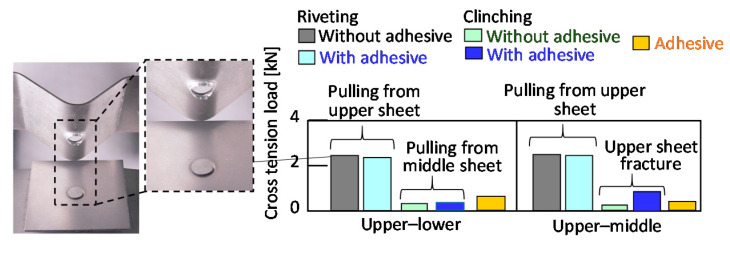
Cross-tension loads of riveted and clinched joints for the upper aluminium alloy sheet.

The cross-tension loads of joints are summarized in Figure 30. The cross-tension loads of joints in the each joining process are smaller than the tension-shear loads in Figure 29. The loads of the riveted joints are higher than that of the adhesive joints because of the low peeling strength of the adhesive in both the upper–lower and the upper–middle sheets. The loads of the clinched joints with and without the adhesive are smaller than those of the riveted joints.

### 4.3. Lower Aluminium Alloy Sheet

**Figure 31:**
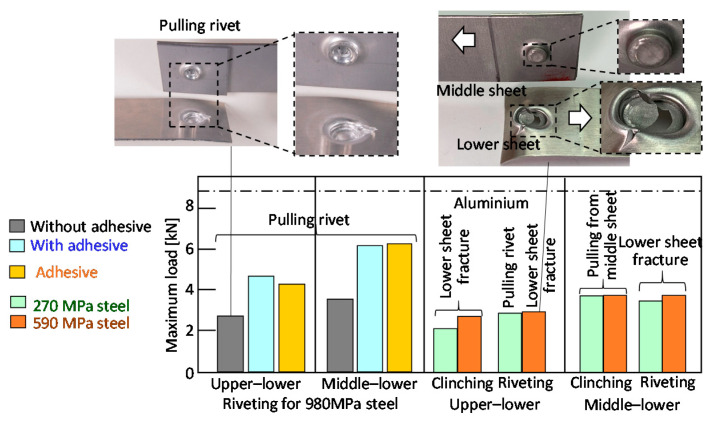
Tension-shearing loads of riveted and clinched joints for the lower aluminium alloy sheet.

The tension-shearing loads of the joints are summarized in Figure 31. In the clinched sheets, the 270 MPa and 590 MPa steel sheets are shown instead of the 980 MPa steel sheets. In the riveted sheets, the tendency is similar to that in Figure 29, i.e., the loads of the adhesive joint and the riveted joint with the adhesive are higher than those without the adhesive. The loads of the clinched sheets in both the upper–lower and middle–lower sheets are similar to those of the riveted sheets because of the soft and thin lower aluminium alloy sheet.

**Figure 32:**
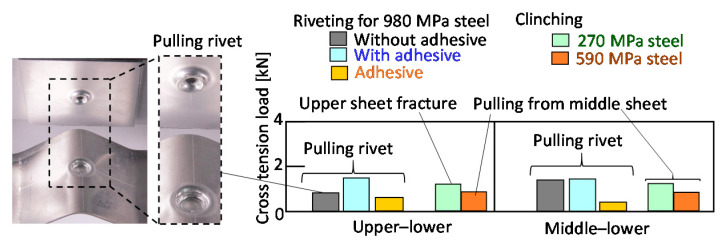
Cross-tension loads of riveted and clinched joints for the lower aluminium alloy sheet.

The cross-tension loads of the joints are summarized in Figure 32. In the clinched sheets, the 270 MPa and 590 MPa steel sheets are shown instead of the 980 MPa steel sheets. The cross-tension loads of the joints are smaller than the tension-shear loads in Figure 31. The loads of the riveted joint in both the upper–lower and the middle–lower sheets are higher than those of the adhesive joint because of the low strength for peeling. The load in the upper–lower sheets of the riveted joint with the adhesive is similar to the load in the middle–lower sheets with the adhesive. Although the loads of the clinched joints are the same or smaller than those of the riveted joints, they are higher than those of the adhesive joints.

## 5. Conclusions

In this study, one 1 mm thick 5000 series aluminium alloy sheet and two 1 mm thick 980 MPa grade cold rolled ultra-high strength steel sheets were joined by self-pierce riveting and mechanical clinching. The joinabilities for the combination of the aluminium and steel sheets in both processes were investigated for different die shapes in the experiment and finite element simulation, and then the strengths of the riveted and clinched joints were compared in the experiment. The results are summarized as follows:

1 The three sheets in both the sheet configurations were successfully joined by self-pierce riveting with the optimum die and rivet.
2 The three sheets in the configuration of the upper aluminium alloy sheet were successfully joined by mechanical clinching with the optimum die, whereas the three sheets in the configuration of the lower aluminium alloy sheet were not joined with the modification of the die shape.
3 In mechanical clinching in the configuration of the lower aluminium alloy sheet, the 590 MPa and 270 MPa steel sheets instead of the 980 MPa steel sheets were joined with the optimum die.
4 Because the tension-shearing load of the adhesive was the highest, the loads of the clinched and riveted joints with the adhesive were similar to the loads of the adhesive.
5 Because the cross-tension load of the adhesive was small, the load increment of the joint with the adhesive was little.

## Figures and Tables

**Table 1:** Material properties of sheets.

Sheet	Sheet Thickness (mm)	Tensile Strength (MPa)	Elongation (%)	Reduction in Area (%)
5000 series aluminium alloy	1.05	275	25	65
980 MPa steel	1.05	1002	14	35

## References

[1] Sakiyama T., Naito Y., Miyazaki Y., Nose T., Murayama G., Saita K., Oikawa H. (2013). Dissimilar Metal Joining Technologies for Steel Sheet and Aluminum Alloy Sheet in Auto Body. Nippon Steel Tech. Rep..

[2] Meschut G., Janzen V., Olfermann T. (2014). Innovative and Highly Productive Joining Technologies for Multi-Material Lightweight Car Body Structures. J. Mater. Eng. Perform..

[3] He X., Pearson I., Young K. (2008). Self-pierce riveting for sheet materials: State of the art. J. Mater. Process. Technol..

[4] Barnes T., Pashby I. (2000). Joining techniques for aluminium spaceframes used in automobiles. J. Mater. Process. Technol..

[5] Porcaro R., Hanssen A., Langseth M., Aalberg A. (2006). Self-piercing riveting process: An experimental and numerical investigation. J. Mater. Process. Technol..

[6] Atzeni E., Ippolito R., Settineri L. (2009). Experimental and numerical appraisal of self-piercing riveting. CIRP Ann..

[7] Casalino G., Rotondo A., Ludovico A. (2008). On the numerical modelling of the multiphysics self piercing riveting process based on the finite element technique. Adv. Eng. Softw..

[8] Abe Y., Kato T., Mori K. Joining of Aluminium Alloy Sheets by Aluminium Alloy Self-Piercing Rivet. Proceedings of the 2nd International Conference on New Forming Technology.

[9] Hoang N.-H., Porcaro R., Langseth M., Hanssen A. (2010). Self-piercing riveting connections using aluminium rivets. Int. J. Solids Struct..

[10] Karim A., Jeong T.-E., Noh W., Park K.-Y., Kam D.-H., Kim C., Nam D.-G., Jung H., Park Y.-D. (2020). Joint quality of self-piercing riveting (SPR) and mechanical behavior under the frictional effect of various rivet coatings. J. Manuf. Process..

[11] Cai W., Wang P., Yang W. (2005). Assembly dimensional prediction for self-piercing riveted aluminum panels. Int. J. Mach. Tools Manuf..

[12] Porcaro R., Hanssen A., Langseth M., Aalberg A. (2006). The behaviour of a self-piercing riveted connection under quasi-static loading conditions. Int. J. Solids Struct..

[13] Atzeni E., Ippolito R., Settineri L. (2007). FEM Modeling of Self-Piercing Riveted Joint. Key Eng. Mater..

[14] Wood P., Schley C.A., Williams M., Rusinek A. (2011). A model to describe the high rate performance of self-piercing riveted joints in sheet aluminium. Mater. Des..

[15] Abe Y., Kato T., Mori K. (2006). Joinability of aluminium alloy and mild steel sheets by self piercing rivet. J. Mater. Process. Technol..

[16] Han L., Chrysanthou A. (2008). Evaluation of quality and behaviour of self-piercing riveted aluminium to high strength low alloy sheets with different surface coatings. Mater. Des..

[17] Ma Y., Lou M., Li Y., Lin Z. (2018). Effect of rivet and die on self-piercing rivetability of AA6061-T6 and mild steel CR4 of different gauges. J. Mater. Process. Technol..

[18] Abe Y., Kato T., Mori K. (2009). Self-piercing riveting of high tensile strength steel and aluminium alloy sheets using conventional rivet and die. J. Mater. Process. Technol..

[19] Mori K., Kato T., Abe Y., Ravshanbek Y. (2006). Plastic Joining of Ultra High Strength Steel and Aluminium Alloy Sheets by Self Piercing Rivet. CIRP Ann..

[20] Uhe B., Kuball C.-M., Merklein M., Meschut G. (2020). Improvement of a rivet geometry for the self-piercing riveting of high-strength steel and multi-material joints. Prod. Eng..

[21] Bouchard P., Laurent T., Tollier L. (2008). Numerical modeling of self-pierce riveting—From riveting process modeling down to structural analysis. J. Mater. Process. Technol..

[22] Han L., Thornton M., Shergold M. (2010). A comparison of the mechanical behaviour of self-piercing riveted and resistance spot welded aluminium sheets for the automotive industry. Mater. Des..

[23] Sun X., Khaleel M.A. (2007). Dynamic strength evaluations for self-piercing rivets and resistance spot welds joining similar and dissimilar metals. Int. J. Impact Eng..

[24] Han L., Chrysanthou A., Young K.W. (2007). Mechanical behaviour of self-piercing riveted multi-layer joints under different specimen configurations. Mater. Des..

[25] Abe Y., Kato T., Mori K.-I. (2008). Self-pierce riveting of three high strength steel and aluminium alloy sheets. Int. J. Mater. Form..

[26] Mori K., Abe Y., Kato T., Sakai S. (2010). Self-Pierce Riveting of Three Aluminium Alloy and Mild Steel Sheets. AIP Conference Proceedings.

[27] Peng H., Chen C., Zhang H., Ran X. (2020). Recent development of improved clinching process. Int. J. Adv. Manuf. Technol..

[28] Varis J. (2006). Economics of clinched joint compared to riveted joint and example of applying calculations to a volume product. J. Mater. Process. Technol..

[29] Lee C.-J., Kim J.-Y., Lee S.-K., Ko D.-C., Kim B.-M. (2010). Design of mechanical clinching tools for joining of aluminium alloy sheets. Mater. Des..

[30] Mizushima D., Murakami H. (2010). Effect of Lubricant Viscosity on Peeling Strength of Mechanical Clinching. J. Jpn. Soc. Technol. Plast..

[31] Varis J. (2003). The suitability of clinching as a joining method for high-strength structural steel. J. Mater. Process. Technol..

[32] Varis J. (2002). The suitability of round clinching tools for high strength structural steel. Thin-Walled Struct..

[33] Chen L.-W., Huang J.-M., Hsu Y.-C. (2015). Investigation of the clinching process combines with hot stamping process for high-strength steel sheets. MATEC Web Conf..

[34] Chen L.-W., Cai M.-J. (2018). Development of a hot stamping clinching tool. J. Manuf. Process..

[35] Abe Y., Kishimoto M., Kato T., Mori K.-I. (2010). Mechanical Clinching of Hot-Dip Zinc-Aluminum Alloy Coated Steel Sheets. J. Jpn. Soc. Technol. Plast..

[36] Behrens B.-A., Bouguecha A., Vučetić M., Hübner S., Yilkiran D., Jin Y., Peshekhodov I. (2015). FEA-based optimisation of a clinching process with an open multiple-part die aimed at damage minimisation in CR240BH-AlSi10MnMg joints. MATEC Web Conf..

[37] Abe Y., Mori K., Kato T. (2012). Joining of high strength steel and aluminium alloy sheets by mechanical clinching with dies for control of metal flow. J. Mater. Process. Technol..

[38] Abe Y., Mori K. (2020). Mechanical Clinching Process with Preforming of Lower Sheet for Joining Aluminium and Ultra-High Strength Steel Sheets. Quart. J. Jpn. Weld. Soc..

[39] Wiesenmayer S., Han D., Meschut G., Merklein M. Investigation of the tool wear behaviour in shear-clinching processes during the running-in phase. Proceedings of the 22nd International Esaform Conference on Material Forming: Esaform 2019.

[40] Busse S., Merklein M., Roll K., Ruther M., Zürn M. (2010). Development of a mechanical joining process for automotive body-in-white production. Int. J. Mater. Form..

[41] Lee C.-J., Lee S.-H., Lee J.-M., Kim B.-H., Kim B.-M., Ko D.-C. (2014). Design of hole-clinching process for joining CFRP and aluminum alloy sheet. Int. J. Precis. Eng. Manuf..

[42] Lambiase F., Ko D.-C. (2016). Feasibility of mechanical clinching for joining aluminum AA6082-T6 and Carbon Fiber Reinforced Polymer sheets. Mater. Des..

[43] Kaðèák L., Spiðák E., Kubík R., Mucha J. (2017). Finite Element Calculation of Clinching with Rigid Die of Three Steel Sheets. Strength Mater..

[44] Kaščák L., Spišák E., Majerníková J. (2016). Joining three car body steel sheets by clinching method. Open Eng..

[45] Moroni F. (2018). Fatigue behaviour of hybrid clinch-bonded and self-piercing rivet bonded joints. J. Adhes..

[46] Neugebauer R., Israel M., Mayer B., Fricke H. (2012). Numerical and Experimental Studies on the Clinch-Bonding and Riv-Bonding Process. Key Eng. Mater..

[47] Japanese Industrial Standards Specimen Dimensions and Procedure for Shear Testing Resistance Spot and Embossed Projection Welds, 1999, JIS Z3136. https://www.jisc.go.jp/app/jis/general/GnrJISNumberNameSearchList?show.

[48] Japanese Industrial Standards Specimen Dimensions and Procedure for Cross Tension Testing Resistance Spot and Embossed Projection Welds, 1999, JIS Z3137. https://www.jisc.go.jp/app/jis/general/GnrJISNumberNameSearchList?toGnrJISStandardDetailList.

